# A Compend of Surgery for Students and Physicians

**Published:** 1887-07

**Authors:** 


**A Compend of Surgery for Students and Physicians.** By ORVILLE HORWITZ, B.S., M.D., Demonstrator of Anatomy in Jefferson Medical College. Third edition. Revised and enlarged, with twenty-one illustrations. Philadelphia: P. Blakiston, Son & Co., 1012 Walnut street.

We have found occasion to speak highly of the entire series of Quiz Compends as far as issued. This little work has met with such favor that already a third edition is demanded. The author has improved it in many ways. The chapters on antiseptic surgery, mortification and gangrene, urethrotomy, burns and scalds, venereal diseases, retention of urine and inflammation have been re-written, and brought thoroughly abreast of our present knowledge.

